# Ferroptosis-related biomarkers for diagnosis and mechanistic insights into Alzheimer’s disease

**DOI:** 10.3389/fnagi.2026.1857642

**Published:** 2026-07-23

**Authors:** Mingchen Zhao, Xinlu Sui, Jieming Li, Qi Wang, Yujun Sun, Haojia Zheng, Bin Li

**Affiliations:** 1 First Clinical Medical College, Shandong University of Traditional Chinese Medicine, Jinan, China; 2 The Affiliated Hiser Hospital of Qingdao University, Qingdao, China

**Keywords:** Alzheimer’s disease, ferroptosis, bioinformatics analysis, NFKBIA, ATP6V1E1, SUB1

## Abstract

**Background:**

Alzheimer’s disease (AD) lacks reliable early diagnostic biomarkers and effective disease-modifying therapies. Ferroptosis has been increasingly implicated in AD pathogenesis; however, ferroptosis-related genes with diagnostic and mechanistic relevance remain insufficiently characterized.

**Methods:**

Transcriptomic datasets were obtained from the GEO database and analyzed using differential expression analysis and WGCNA, followed by integration with ferroptosis-related gene sets. Key targets were identified through PPI networks and machine learning approaches, including LASSO regression and random forest. A diagnostic model was constructed and its discriminative performance was assessed across independent datasets by cohort-specific refitting. Functional roles were explored using GSEA, and experimental validation was conducted by qPCR in Aβ-treated PC12 cells. Potential therapeutic compounds were predicted using the CMAP database.

**Results:**

A total of 52 ferroptosis-related candidate genes were identified. Among these, three genes, *NFKBIA*, *ATP6V1E1*, and *SUB1*, were consistently selected as core features by machine learning algorithms. A diagnostic model based on these genes demonstrated strong performance in the training cohort (AUC > 0.8) and reproducible discriminative ability across external datasets, albeit with cohort-dependent variability. Functional analysis suggested that these genes are involved in pathways related to neuroinflammation, lysosomal activity, and oxidative stress, indicating a potential link between ferroptosis and key pathological processes in AD. qPCR validation confirmed the differential expression trends of these genes. In addition, several candidate compounds, including etomoxir and tubastatin A, were predicted to potentially modulate these pathways.

**Conclusion:**

Three ferroptosis-related genes, *NFKBIA*, *ATP6V1E1*, and *SUB1*, were identified in Alzheimer‘s disease and showed consistent discriminative ability across multiple cohorts, with links to neuroinflammation, lysosomal function, and oxidative stress. These findings highlight their potential as candidate biomarkers warranting further investigation, although prospective validation in independent cohorts is required to confirm their diagnostic utility.

## 1 Introduction

Alzheimer’s disease (AD), the most common form of dementia, is a progressive neurodegenerative disorder and a leading cause of cognitive decline in older adult (Scheltens et al., 2016). Its incidence and mortality continue to rise, posing a major public health challenge. It is estimated that 6.9 million Americans aged ≥65 years are affected, a number projected to reach 13.8 million by 2060, with 119,399 deaths recorded in 2021 (Alzheimer's Association, 2024). Clinically, AD leads to progressive loss of memory and functional independence, often accompanied by neuropsychiatric symptoms such as depression, anxiety, and irritability, which further impair quality of life and increase caregiver burden (Sharif et al., 2025; Pelucio et al., 2023). In addition, AD imposes a substantial economic burden, with global costs estimated at USD 14,513 billion (Chen et al., 2024). Improving early detection and identifying effective therapeutic targets remain critical.

Current diagnosis of AD relies primarily on clinical assessment and cognitive testing, which lack specificity and can be confounded by other neurological disorders (Knopman et al., 2021). For example, dementia with Lewy bodies shares overlapping clinical features with AD (Sim et al., 2022). Neuroimaging and fluid biomarkers have improved diagnostic accuracy, including structural MRI for hippocampal atrophy and detection of tau and amyloid-β via cerebrospinal fluid, plasma, and PET (Salta et al., 2023; Morris and Mcdade, 2022). However, these markers typically reflect relatively advanced pathology, limiting their value for early diagnosis. Thus, identifying upstream molecular and genetic alterations is essential.

Genetic and epigenetic dysregulation plays an important role in AD (Schuler et al., 2022; Jayadev, 2022; Xiong et al., 2023). Current therapies mainly provide symptomatic relief and do not halt disease progression, whereas gene-based approaches offer the potential to target underlying mechanisms (Doshi et al., 2024; Wu et al., 2025). Therefore, identifying key regulatory genes is important for both early diagnosis and therapeutic development.

Ferroptosis, an iron-dependent form of regulated cell death driven by lipid peroxidation (Zheng and Conrad, 2025), has been increasingly implicated in AD. Disrupted iron homeostasis and impaired antioxidant systems contribute to oxidative stress and neuronal damage (Feng et al., 2024; Wu et al., 2023). Notably, ferroptosis is closely linked to key pathological features of AD, including iron accumulation, lipid peroxidation, and mitochondrial dysfunction, suggesting a distinct role in disease progression. Targeting ferroptosis-related pathways may therefore provide a mechanistically relevant approach for early intervention. However, the roles of key ferroptosis-related genes in AD remain unclear.

In this study, we identified key ferroptosis-related genes in AD and assessed their discriminative ability and potential mechanistic relevance through integrated analysis and experimental evaluation.

## 2 Methods

### 2.1 Data collection and processing

The AD datasets were obtained from the GEO database (https://www.ncbi.nlm.nih.gov/geo/). Four AD-related datasets (GSE132903, GSE63060, GSE63061, GSE29378) were included in this study. GSE132903 was used for the primary analysis. Meanwhile, the expression of key common genes was validated in GSE63060, GSE63061, and GSE29378. Sample demographics (age, sex, disease stage) for each dataset have been described in the original studies: GSE132903 (Piras et al., 2019), GSE63060 (Sood et al., 2015), GSE63061 (Sood et al., 2015), and GSE29378 (Miller et al., 2013). A summary of sample sizes and tissue sources is provided in Table 1.

**Table 1:** Summary of AD-related GEO datasets used in this study.

Accession number	Platform	Simples (disease/control)	Sample source	Attribute
GSE132903	GPL10558	97/98	Middle temporal gyrus tissue	Train set
GSE63060	GPL6801	145/104	Blood	Validation set
GSE63061	GPL6887	139/135	Blood	Validation set
GSE29378	GPL6947	31/32	Hippocampal tissue	Validation set

### 2.2 Differential expression analysis

Raw expression data from GSE132903 were downloaded from GEO. Probe-level data were mapped to gene symbols using the platform annotation file (GPL10558). When multiple probes corresponded to the same gene, the average expression value was calculated. Data were then log₂-transformed (if raw values were not already on a log scale) and normalized between arrays using the normalizeBetweenArrays function in the limma package. Differential expression analysis between AD and control samples was performed using the limma package (version 4.3.3) with an empirical Bayes moderated t-test. Differentially expressed genes (DEGs) were defined using thresholds of |log₂ fold change| > 0.5 and adjusted *p* value < 0.05 (Benjamini–Hochberg false discovery rate correction). Volcano plots and heatmaps were generated using appropriate R packages.

No batch correction was applied to the primary dataset (GSE132903) as it was analyzed individually. For the validation datasets (GSE63060, GSE63061, GSE29378), each was processed independently following the same normalization procedure; cross-dataset batch correction was not performed, and potential batch effects are acknowledged as a limitation.

### 2.3 Weighted gene co-expression network analysis (WGCNA)

To identify gene modules closely associated with AD, WGCNA was performed. Raw data were filtered, and the soft-thresholding power was determined by evaluating network topology for powers 1–20. A scale-free network was constructed with the optimal power of 15 (scale-free R^2^ > 0.8; stable average connectivity), transforming the expression matrix into an adjacency matrix and then a topological overlap matrix (TOM). Gene dissimilarity was calculated based on TOM, followed by hierarchical clustering. Modules were identified using a dynamic tree-cutting algorithm (minimum module size = 15, deepSplit = 2) and merged if module eigengene dissimilarity < 0.8. Correlations between module eigengenes and clinical traits were calculated to select modules significantly associated with AD. The online Venn diagram tool jvenn was used to intersect WGCNA module genes with DEGs.

### 2.4 Functional enrichment analysis

To explore the biological relevance of intersecting genes, Gene Set Enrichment Analysis (GSEA), Gene Ontology (GO), and Kyoto Encyclopedia of Genes and Genomes (KEGG) pathway enrichment analyses were performed. A pre-ranked gene list based on log₂FC values was used for GSEA, with the top five up- and down-regulated pathways visualized using the enrichplot package. GO and KEGG enrichment analyses were conducted via Metascape, and results were visualized using the Microbiome Informatics Network Platform.

### 2.5 Construction of the protein–protein interaction (PPI) network

We obtained the initial ferroptosis-related gene set from GeneCards using the keyword “ferroptosis,” indicating strong association based on literature curation and text mining. The resulting gene set was then sequentially filtered using DEGs, WGCNA, PPI, and machine learning to remove weakly associated genes. Three gene sets were intersected: DEGs from differential expression analysis, WGCNA brown module genes, and ferroptosis-related genes retrieved from the GeneCards database. The STRING database was used to construct a PPI network with a minimum interaction score of 0.400. Cytoscape (v3.8.0) and the CytoNCA plugin were used to visualize and analyze the PPI network.

### 2.6 Feature selection based on LASSO regression and random forest

Core candidate genes were identified using two machine learning methods: LASSO regression and random forest. LASSO regression was performed using the glmnet package (family = binomial, nfolds = 10) to select features with non-zero coefficients. Based on LASSO results, a random forest model was constructed using the randomForest package (ntree = 2000), with the optimal number of trees determined by the out-of-bag (OOB) error rate. Gene importance was evaluated using Mean Decrease in Gini, and highly contributing genes were selected as core candidates.

### 2.7 Logistic regression-based diagnostic model

A logistic regression model was constructed using glmnet and glm packages. Stepwise regression was applied to determine the optimal combination of core genes. Multicollinearity among the selected genes was assessed using the variance inflation factor (VIF). The model was visualized as a nomogram using the rms package, and predictive performance was assessed using calibration curves and the C-index. Receiver operating characteristic (ROC) and decision curve analyses were performed using pROC and rmda packages to evaluate diagnostic accuracy (AUC) and clinical applicability.

### 2.8 Single-gene GSEA

To investigate the functional roles of core genes at the pathway level, single-gene GSEA was performed. Samples were divided into high- and low-expression groups based on the median expression of each gene. Differential expression analysis was conducted using limma, and GSEA-KEGG enrichment analysis was performed using clusterProfiler. Ridge plots were generated using the ridgeplot function in enrichplot to visualize enrichment scores and pathway distributions.

### 2.9 External validation and experimental validation

Expression levels of ferroptosis-related core genes were validated in GSE63060, GSE63061, and GSE29378. Each dataset underwent background correction, normalization, and log₂ transformation using limma, after which target gene expression was extracted. For each validation dataset, independent-samples t-tests were performed to compare expression levels between AD and control groups. When Levene’s test indicated unequal variances (*p* < 0.05), Welch’s t-test (not assuming equal variances) was used. The mean difference (AD minus control) was reported as the log₂ fold change. To assess the generalizability of the three-gene signature to independent datasets, we separately fitted logistic regression models using *NFKBIA*, *ATP6V1E1*, and *SUB1* as predictors on each of the external validation datasets (GSE63060, GSE63061, and GSE29378). The riskScore was defined as the linear combination of the three genes, and the area under the receiver operating characteristic curve (AUC) was calculated for each dataset.

To explore the relationship between the three core genes and key ferroptosis regulators, Pearson correlation analysis was performed using the expression data of *NFKBIA*, *ATP6V1E1*, *SUB1*, *GPX4*, and *ACSL4* in the GSE132903 dataset. Correlation coefficients and two-tailed *p*-values were calculated.

For experimental validation, PC12 cells were used to model AD-like neuronal injury via Aβ treatment. Briefly, PC12 cells were cultured in DMEM supplemented with 10% fetal bovine serum and differentiated with nerve growth factor. Cells were then treated with Aβ₁-₄₂ (10 μM) for 24 h to induce AD-like pathological conditions. Total RNA was extracted using TRIzol, and cDNA was synthesized using a reverse transcription kit. Quantitative real-time PCR (qPCR) was performed using SYBR Green Master Mix on a QuantStudio 6 system. Gene expression was normalized to *GAPDH*, and relative expression levels were calculated using the 2^^-ΔΔCt^ method.

### 2.10 Drug prediction

To explore potential therapeutic strategies targeting ferroptosis in AD, the 52 ferroptosis-related intersecting genes (24 up-regulated, 28 down-regulated) were divided into two separate gene sets (up-regulated and down-regulated) and submitted to Connectivity Map (CMAP) via the CLUE platform (https://clue.io/). Normalized connectivity scores (NCS) and adjusted *p*-values were used to evaluate potential compounds. A negative NCS suggests that a compound may reverse the input disease signature, with larger absolute values indicating stronger predicted effects. The NCS ranges from −1 to 1. A negative value indicates that a compound may reverse the input disease signature, while a positive value indicates a similar expression pattern to the disease. The absolute value reflects the strength of the connection.

## 3 Results

### 3.1 Differential expression analysis and identification of key AD-related modules

**Figure 1:**
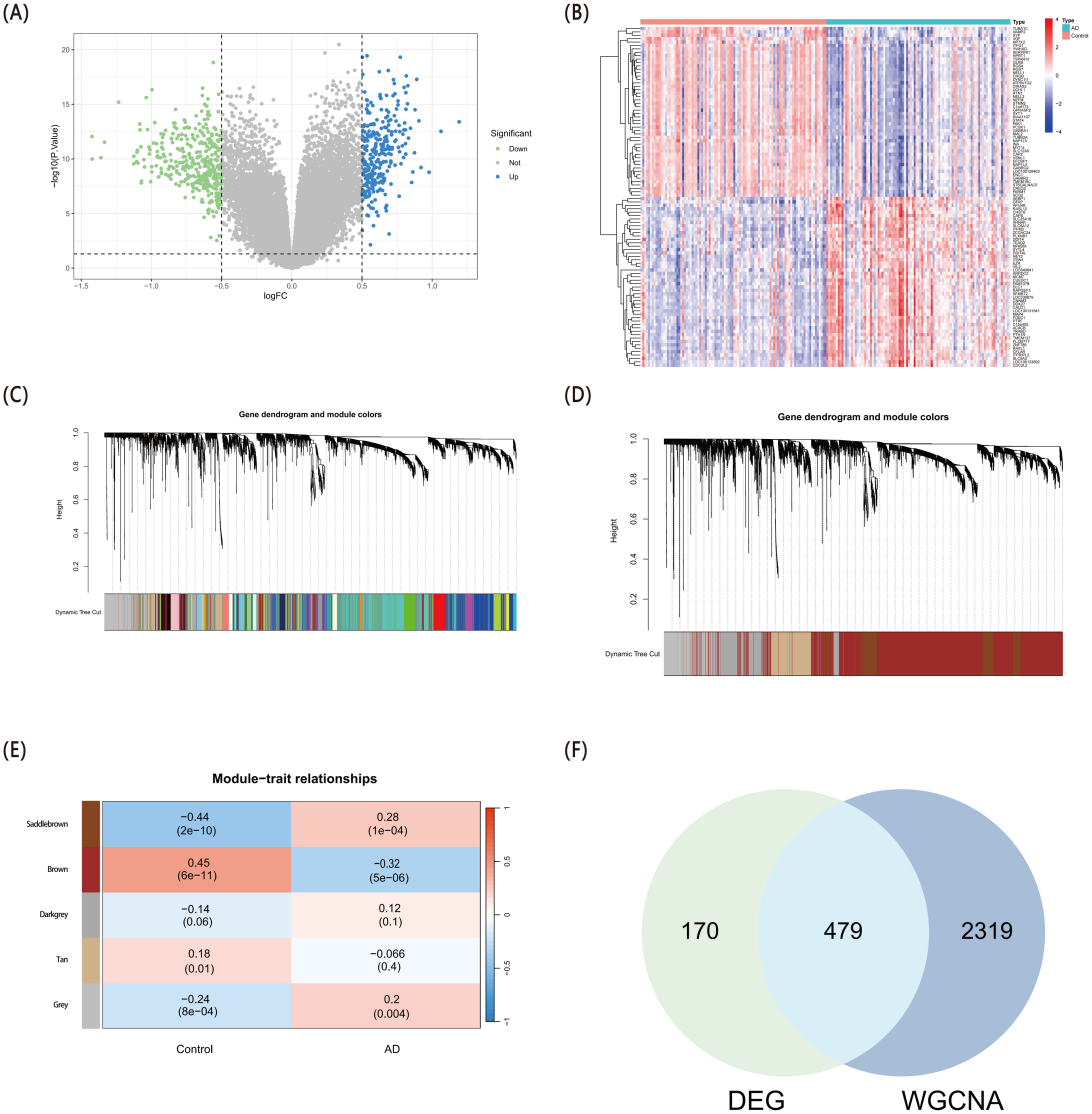
Differential expression analysis and identification of AD-related co-expression modules. **(A)** Volcano plot of differentially expressed genes between AD and control samples. **(B)** Heatmap showing expression patterns of the DEGs. **(C)** Dendrogram of all genes clustered into modules by WGCNA. **(D)** Merged co-expression modules. **(E)** Correlation between module eigengenes and clinical traits. **(F)** Venn diagram showing the intersection between DEGs and the brown module genes.

Differential expression analysis of middle temporal gyrus tissue from AD patients and normal controls identified a total of 649 differentially expressed genes (DEGs). Among these, 283 genes were significantly upregulated, including *RBPJ*, *MCM7*, *NFKBIA*, *PLXNB1*, and *SLC5A3*, while 366 genes were significantly downregulated, including *NDUFAB1*, *HSPB3*, *NAP1L5*, *VGF*, and *GABRA1*. The overall distribution and expression patterns of these DEGs are shown in a volcano plot (Figure 1A) and heatmap (Figure 1B).

To investigate the relationship between DEGs and AD, weighted gene co-expression network analysis was performed. Initial clustering identified multiple gene modules (Figure 1C). After merging redundant modules, five stable modules were obtained (Figure 1D). Correlation analysis between module eigengenes and clinical traits revealed significant associations with the brown, darkgrey, grey, saddlebrown, and tan modules (Figure 1E). The brown module, which included genes such as *SYT1*, *CHGB*, *RGS4*, *SERPINI1*, and *DYNC1I1*, showed a correlation coefficient of 0.32 with the AD group and 0.45 with the healthy group. A total of 2,798 genes from the brown module were intersected with the DEGs (Figure 1F) for subsequent analyses.

### 3.2 Functional enrichment analysis

**Figure 2:**
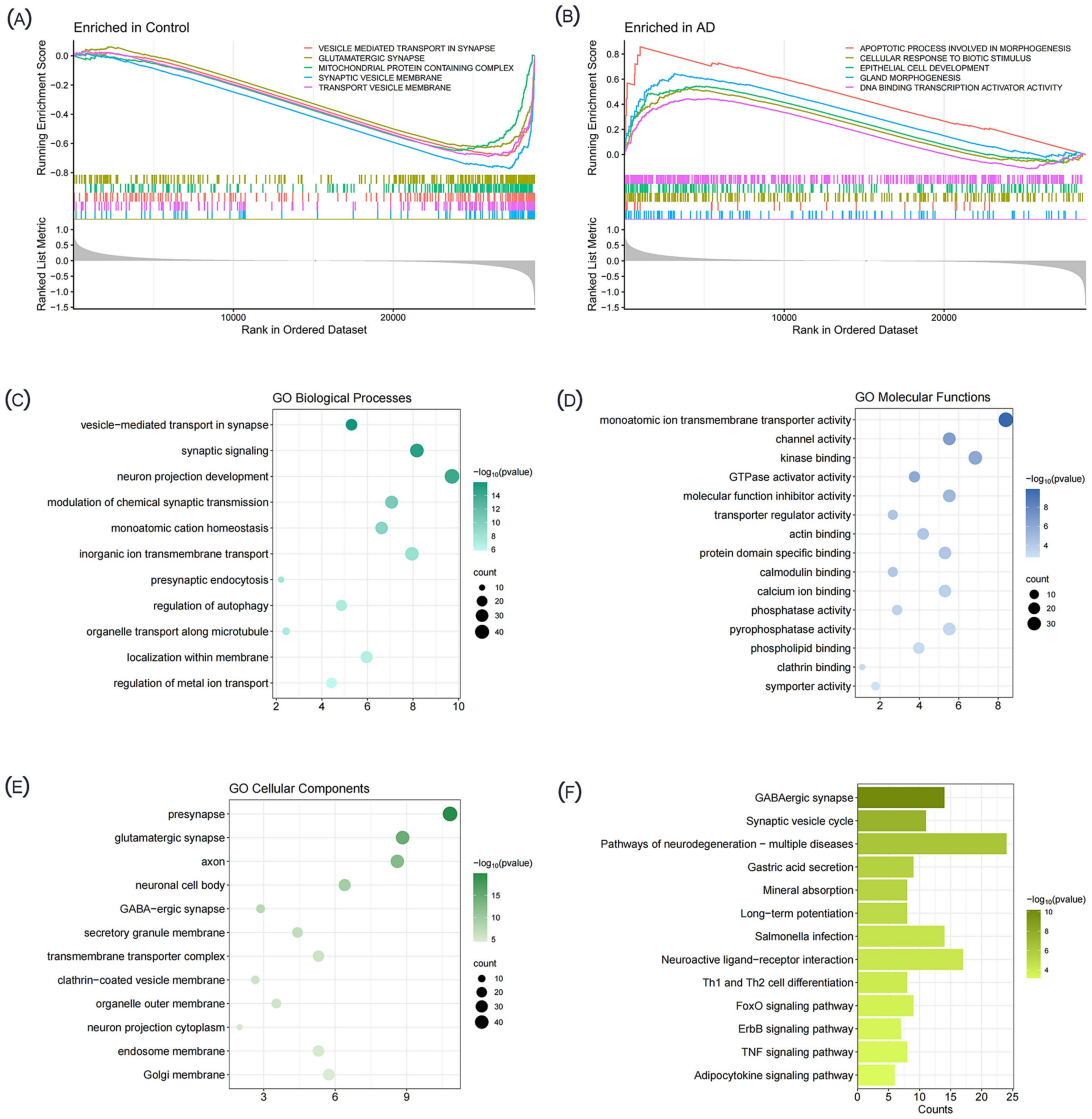
Functional enrichment analysis of intersecting genes. **(A,B)** GSEA showing enriched pathways in control and AD groups. **(C–E)** GO enrichment analysis for biological process, molecular function, and cellular component. **(F)** KEGG pathway enrichment analysis.

GSEA comparing AD and control samples revealed distinct enrichment patterns. Genes in the control group were enriched in vesicle-mediated transport at the synapse, glutamatergic synapse, mitochondrial protein-containing complex, synaptic vesicle membrane, and transport vesicle membrane, whereas genes in the AD group were enriched in apoptotic processes involved in morphogenesis, cellular response to biotic stimulus, epithelial cell development, gland morphogenesis, and DNA-binding transcription activator activity (Figures 2A,B). GO and KEGG enrichment analyses of intersecting genes from differential expression and WGCNA identified enrichment in monoatomic cation homeostasis, inorganic ion transmembrane transport, regulation of autophagy, monoatomic ion transmembrane transporter activity, phospholipid binding, transporter regulator activity, presynapse, endosome membrane, transmembrane transporter complex, mineral absorption, pathways of neurodegeneration in multiple diseases, and FoxO signaling pathway (Figures 2C–F).

### 3.3 Construction of the protein–protein interaction network

**Figure 3:**
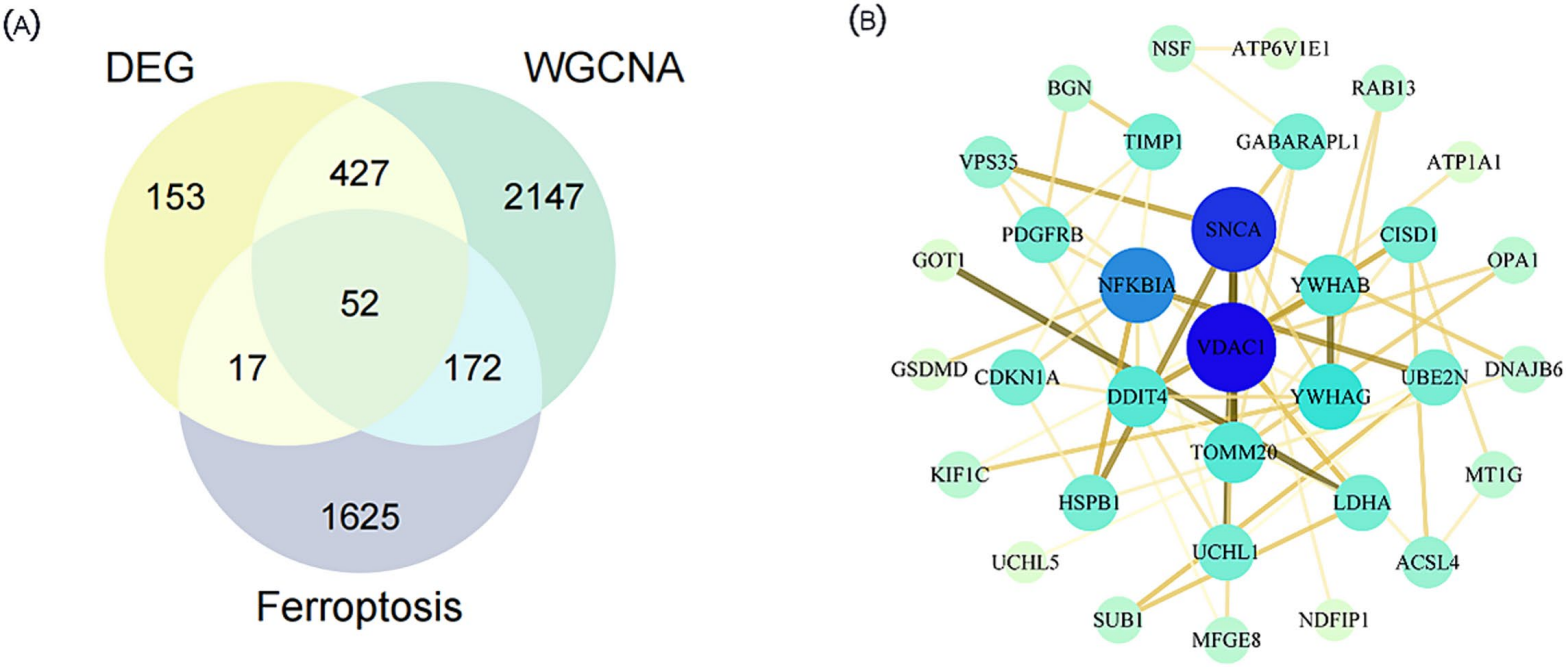
Protein–protein interaction network of ferroptosis-related intersecting genes. **(A)** Network visualization of 52 intersecting genes. **(B)** Network highlighting the most highly connected hub genes.

A protein–protein interaction network was constructed using the 52 intersecting genes derived from differential expression analysis, WGCNA, and the ferroptosis gene set. The network contained 51 nodes and 58 edges, with isolated nodes excluded from the visualization. The most highly connected hub genes were *VDAC1*, *SNCA*, and *NFKBIA*. The network exhibited an average node degree of 2.27 and an average local clustering coefficient of 0.361. PPI enrichment analysis showed significant biological connectivity with a *p* value of 1.2e-08, indicating that the observed interactions are substantially more frequent than expected by chance (Figure 3).

### 3.4 Feature selection based on LASSO regression and random forest

**Figure 4:**
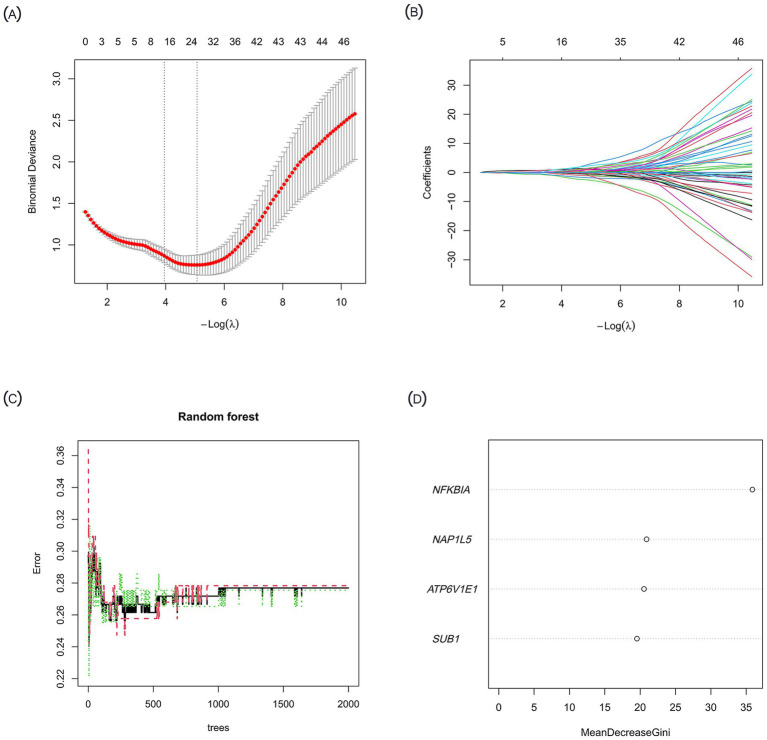
Feature selection using LASSO regression and random forest. **(A,B)** LASSO coefficient profiles and cross-validation to identify candidate genes. **(C,D)** Random forest analysis ranking the importance of candidate genes and selecting core genes.

LASSO regression analysis identified 27 candidate genes from the 52 ferroptosis-related differentially expressed genes. These genes were further evaluated using random forest analysis, which selected four genes, *NFKBIA*, *NAP1L5*, *ATP6V1E1*, and *SUB1*, as candidate targets for subsequent analyses (Figure 4). During subsequent stepwise logistic regression (section 3.5), *NAP1L5* was automatically excluded because its regression coefficients were unstable across cross-validation folds, indicating poor discriminative reliability. Therefore, the final diagnostic model comprised *NFKBIA*, *ATP6V1E1*, and *SUB1.*

### 3.5 Construction of a logistic regression-based diagnostic model

**Figure 5:**
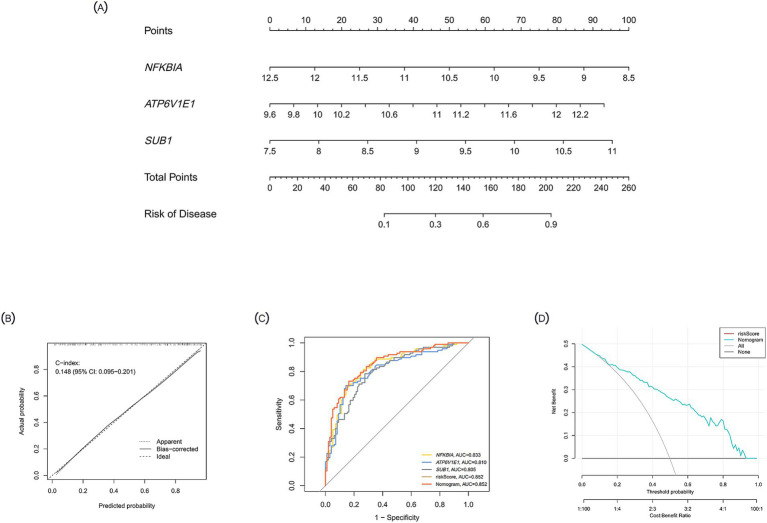
Logistic regression-based diagnostic model for AD. **(A)** Nomogram integrating three key genes. **(B)** Calibration curve assessing model performance. **(C)** ROC curves for individual genes and combined model. **(D)** Decision curve analysis evaluating clinical applicability.

A logistic regression model for AD risk prediction was constructed using three key genes, *NFKBIA*, *ATP6V1E1*, and *SUB1*. *ATP6V1E1* (O*R* = 0.298, 95% CI: 0.105–0.804, *p* = 0.019) and *SUB1* (O*R* = 0.371, 95% CI: 0.138–0.928, *p* = 0.040) showed significant negative correlations with disease risk, while *NFKBIA* (O*R* = 2.482, 95% CI: 0.933–6.890, *p* = 0.073) did not reach statistical significance but exhibited an odds ratio greater than 1. Multicollinearity among the three selected genes was assessed using the variance inflation factor (VIF), and all VIF values were below 5 (*NFKBIA*: 3.819, *ATP6V1E1*: 2.451, *SUB1*: 2.504), indicating no significant multicollinearity. A nomogram, calibration curve, ROC curve, and decision curve analysis were generated to evaluate the model. Among individual genes, the AUC values were 0.833 for *NFKBIA*, 0.810 for *ATP6V1E1*, and 0.805 for *SUB1*. The combined model, including the multi-gene risk score and nomogram, achieved an AUC of 0.852 (Figure 5).

### 3.6 Single-gene GSEA

**Figure 6:**
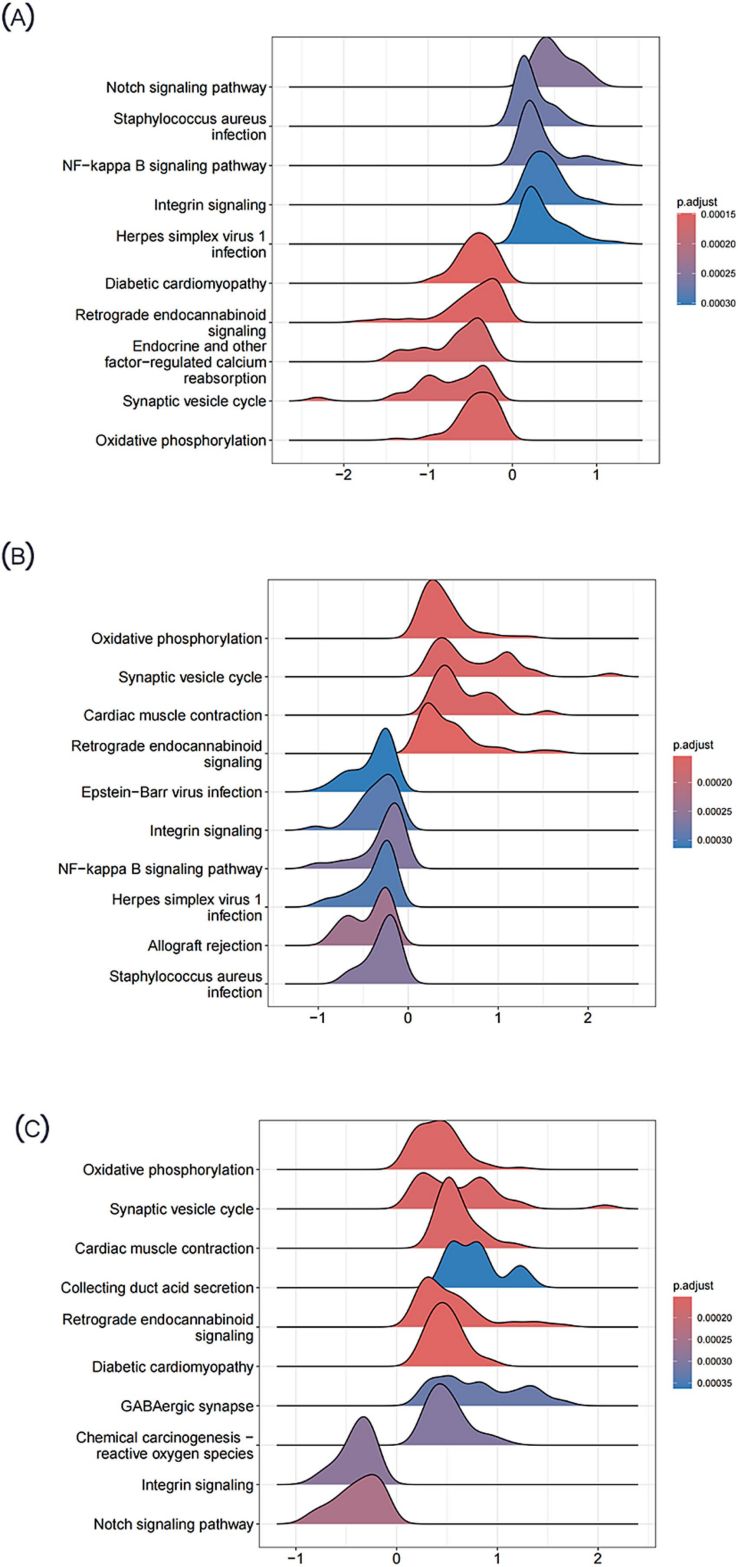
Single-gene GSEA for *NFKBIA*, *ATP6V1E1*, and *SUB1*. Enriched pathways associated with each gene in AD samples.

Single-gene GSEA was performed for *NFKBIA*, *ATP6V1E1*, and *SUB1*. *NFKBIA* was enriched in NF-kappa B signaling, oxidative phosphorylation, synaptic vesicle cycle, and Notch signaling (Figure 6A). *ATP6V1E1* was enriched in oxidative phosphorylation, synaptic vesicle cycle, NF-kappa B signaling, and integrin signaling (Figure 6B). *SUB1* was enriched in oxidative phosphorylation, synaptic vesicle cycle, chemical carcinogenesis-reactive oxygen species, integrin signaling, and Notch signaling (Figure 6C).

### 3.7 External validation and experimental validation

**Figure 7:**
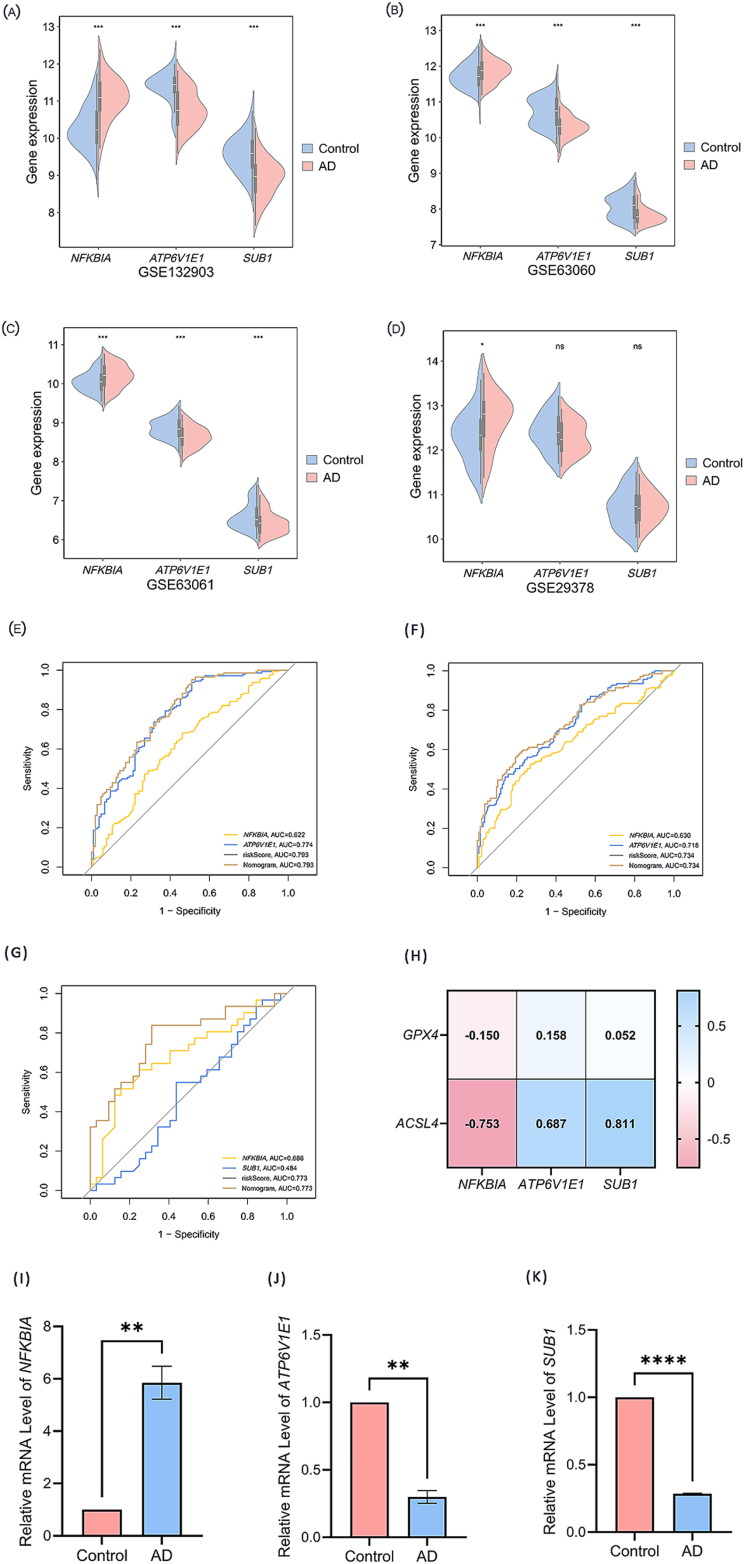
External validation and qPCR validation of core genes. **(A–D)** Expression levels of *NFKBIA*, *ATP6V1E1*, and *SUB1* in the GSE132903, GSE63060, GSE63061, and GSE29378 datasets. **(E–G)** ROC curves of the logistic regression model in the three external validation datasets. **(H)** Pearson correlation heatmap between the three core genes and the ferroptosis regulators *GPX4* and *ACSL4*. (I–K) qPCR validation of *NFKBIA*, *ATP6V1E1*, and *SUB1* expression in Aβ-treated PC12 cells compared with control cells. Data are presented as mean ± SD (error bars). Ns denotes not significant, *p < 0.05, **p < 0.01, ***p < 0.001, ****p < 0.0001; all significance levels indicate comparisons with the control group.

The expression levels of *NFKBIA*, *ATP6V1E1*, and *SUB1* were consistently detected across the GSE132903, GSE63060, GSE63061, and GSE29378 datasets (Figures 7A–D). *NFKBIA* was upregulated in AD samples compared with controls, whereas *ATP6V1E1* and *SUB1* were downregulated. Statistical significance is indicated as *p* < 0.05, *p* < 0.01, *p* < 0.001, *p* < 0.0001, and ns denotes not significant. The log₂ fold changes of *NFKBIA*, *ATP6V1E1*, and *SUB1* in each validation dataset (GSE63060, GSE63061, and GSE29378) are summarized in Supplementary Table S1. These findings support the reproducibility of these three genes across multiple cohorts. We additionally fitted logistic regression models using the three genes on these external validation datasets, and the riskScore AUC values as well as the ROC curves were obtained (Figures 7E–G).

Pearson correlation analysis was performed to explore the relationships between the three core genes (*NFKBIA*, *ATP6V1E1*, *SUB1*) and the ferroptosis regulators *GPX4* and *ACSL4*. As shown in the heatmap (Figure 7H), *NFKBIA* showed significant negative correlations with *ACSL4* (*r* = −0.753, *p* < 0.01) and *GPX4* (*r* = −0.150, *p* < 0.05). *ATP6V1E1* exhibited significant positive correlations with *ACSL4* (*r* = 0.687, *p* < 0.01) and *GPX4* (*r* = 0.158, *p* < 0.05). *SUB1* was strongly positively correlated with *ACSL4* (*r* = 0.811, *p* < 0.01) but not significantly correlated with *GPX4* (*r* = 0.052, *p* = 0.469).

Consistent with the transcriptomic analyses, qPCR validation in Aβ-treated PC12 cells showed a significant increase in *NFKBIA* expression and significant decreases in *ATP6V1E1* and *SUB1* expression compared with control cells (Figures 7I–K).

### 3.8 Drug prediction

**Figure 8:**
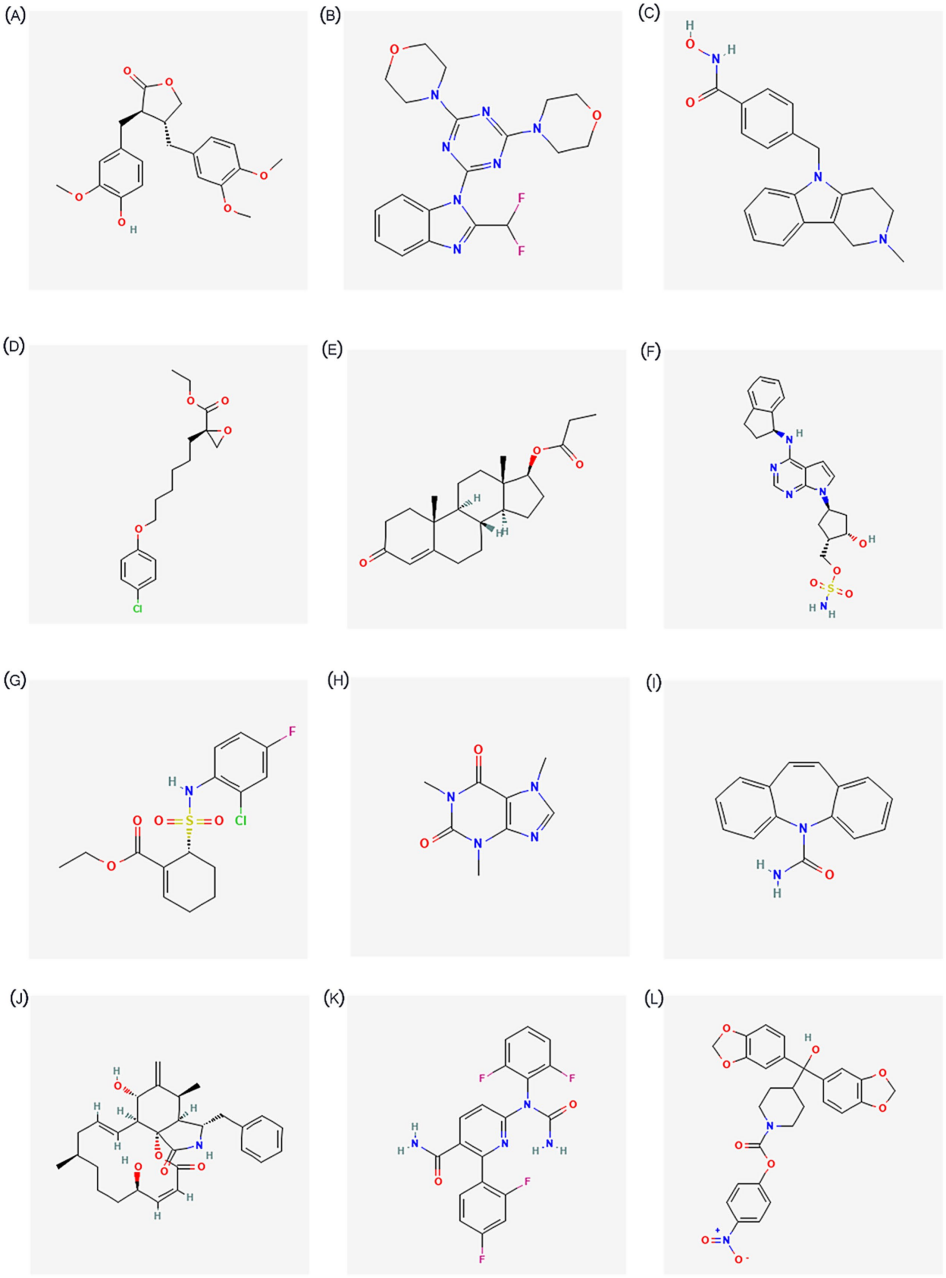
Chemical structures of the 12 predicted compounds. **(A)** arctigenin, **(B)** ZSTK-474, **(C)** tubastatin-a, **(D)** etomoxir, **(E)** testosterone-propionate, **(F)** MLN-4924, **(G)** resatorvid, **(H)** caffeine, **(I)** carbamazepine, **(J)** BRD-K26664453, and **(K)** VX-702, **(L)** JZL-184.

Based on CMAP analysis, the top 12 compounds with the strongest negative normalized connectivity scores were identified, including Etomoxir, Tubastatin-A, Resatorvid, Carbamazepine, Arctigenin, Testosterone propionate, ZSTK-474, Caffeine, MLN-4924, BRD-K26664453, VX-702, and JZL-184 (Figures 8A–L; Table 2). All compounds met the extreme statistical significance criterion with -log10(FDR) greater than 15.

**Table 2:** Top 12 predicted compounds from CMAP analysis.

Rank	Predicted compound	Chemical formula	Predicted compound	-log₁₀ (FDR)
1	Arctigenin	C21H24O6	−1.8118	15.6536
2	ZSTK-474	C19H21F2N7O2	−1.7685	15.6536
3	Tubastatin-a	C20H21N3O2	−1.7685	15.6536
4	Etomoxir	C17H23ClO4	−1.7473	15.6536
5	Testosterone-propionate	C22H32O3	−1.7431	15.6536
6	MLN-4924	C21H25N5O4S	−1.7187	15.6536
7	Resatorvid	C15H17ClFNO4S	−1.6798	15.6536
8	Caffeine	C8H10N4O2	−1.6698	15.6536
9	Carbamazepine	C15H12N2O	−1.6691	15.6536
10	BRD-K26664453	C29H37NO5	−1.656	15.6536
11	VX-702	C19H12F4N4O2	−1.6093	15.6536
12	JZL-184	C27H24N2O9	−1.6068	15.6536

## 4 Discussion

AD is a progressive neurodegenerative disorder characterized by memory impairment, cognitive decline, and loss of functional autonomy (Zhang et al., 2024). Despite decades of research, no therapies effectively reverse or halt disease progression, and early diagnostic approaches based on clinical symptoms or imaging often lack sufficient sensitivity and specificity. This gap emphasizes the need for molecular biomarkers that may facilitate early detection and inform therapeutic strategies. Recent evidence indicates that ferroptosis, a form of iron-dependent cell death triggered by lipid peroxidation, contributes to AD pathogenesis. Iron accumulation has been observed in neuroinflammatory plaques, reaching levels up to 2.8 times higher than normal (Liu et al., 2019), and may arise from neuronal degeneration rather than merely amyloid-beta deposition (Streit et al., 2025). Dysregulation of iron homeostasis, impaired antioxidant defenses, and lipid peroxidation collectively create a cellular environment that favors ferroptotic neuronal death, linking ferroptosis to key pathological features of AD, including oxidative stress, synaptic dysfunction, and neuronal loss (Lei et al., 2025).

Mechanistically, these genes converge on critical processes implicated in ferroptosis and AD pathology. *NFKBIA* encodes IκBα, a central regulator of NF-κB signaling. In the AD brain, chronic stimuli such as Aβ deposition and tau hyperphosphorylation can lead to IκBα degradation, disinhibiting NF-κB and promoting sustained neuroinflammation. This inflammatory milieu increases ROS production and lipid peroxidation, creating conditions that favor ferroptosis (Chen Y. et al., 2023). Based on our transcriptomic data combined with WGCNA, PPI network analysis, and machine learning algorithms, we identified *NFKBIA*, *ATP6V1E1*, and *SUB1* as three genes closely associated with both AD and ferroptosis. We chose Aβ-treated PC12 cells as our *in vitro* model. This system has been widely used in neurodegeneration studies, and recent work has confirmed that Aβ exposure in PC12 cells induces ferroptosis-related changes, including lipid peroxidation accumulation and *GPX4* downregulation (Zhang et al., 2022; Li et al., 2026), making it a suitable platform for evaluating whether these candidate genes modulate ferroptosis in the context of AD-related toxicity. In our in vitro experiments, Aβ-treated PC12 cells showed significant upregulation of *NFKBIA* and significant downregulation of *ATP6V1E1* and *SUB1* compared with control cells.

*ATP6V1E1* encodes a subunit of the V-ATPase proton pump, which maintains lysosomal and endosomal acidity. Its downregulation in AD may impair lysosomal degradation of Aβ and tau, disrupt intracellular iron handling, and exacerbate oxidative stress, all of which enhance neuronal vulnerability to ferroptosis (Terman and Kurz, 2013). Although direct experimental evidence linking *ATP6V1E1* to ferroptosis is currently lacking, a well-established mechanistic framework supports such a connection. Lysosomal acidification dysfunction is a recognized driver of iron dyshomeostasis and ferroptosis (Rizzollo et al., 2021). Moreover, other V-ATPase subunits (e.g., *ATP6V0A1*) have been shown to regulate lysosomal acidification and induce ferritinophagy-dependent ferroptosis (Gu et al., 2026). By analogy, the downregulation of *ATP6V1E1* in AD likely impairs lysosomal acidification, leading to iron overload and lipid peroxidation, thereby promoting neuronal ferroptosis.

*SUB1*, a transcriptional co-regulator, modulates stress-response genes and antioxidant defense mechanisms, including pathways downstream of NRF2. Reduced *SUB1* expression may weaken these defenses, leaving neurons unable to counteract iron-induced oxidative stress and lipid peroxidation, further promoting ferroptosis (Yan et al., 2023). Additionally, *SUB1* (also known as PC4) has been shown to interact with *NUP214* and repress the transcription of pro-ferroptotic genes, including *ALOX15* and *HMOX1*, thereby protecting cells from lipid peroxidation and ferroptosis (Chen Z. et al., 2023). Therefore, downregulation of *SUB1* in AD may relieve this transcriptional repression, leading to increased expression of these pro-ferroptotic factors and enhanced neuronal vulnerability to ferroptosis. Together, these genes form a molecular network integrating iron metabolism, oxidative stress, and inflammation, which likely underpins the ferroptotic susceptibility of neurons in AD. It should be noted that the PPI network and the diagnostic model address different biological and analytical questions. During our analysis, we observed that the hub genes identified in the PPI network were not fully consistent with the features selected by the LASSO-logistic regression model. The PPI network identifies physically interacting hub genes (e.g., *VDAC1*, *SNCA*) from a topological perspective, reflecting their potential centrality in protein interaction systems. In contrast, LASSO and logistic regression select features optimized for discriminating AD from controls based on statistical contribution to classification performance. Therefore, a hub gene in the interaction network is not necessarily a strong discriminative marker, and vice versa.

To explore potential interventions, CMAP analysis predicted several compounds that may reverse the ferroptosis gene signature in AD. Etomoxir, an inhibitor of *CPT1*, may modulate mitochondrial energy metabolism and lipid homeostasis, indirectly mitigating ferroptosis (Schlaepfer and Joshi, 2020). Tubastatin-A, a selective *HDAC6* inhibitor, can improve microtubule stability and reduce neuroinflammation, potentially interfering with the *NFKBIA*/NF-κB-mediated ferroptotic cascade (Sui et al., 2020). Resatorvid, a *TLR4* antagonist, suppresses upstream inflammatory signaling, thereby alleviating neuroinflammation-driven ferroptosis (Engelmann et al., 2022). Other compounds such as Arctigenin, Testosterone propionate, Carbamazepine, and JZL-184 offer additional avenues for modulating oxidative stress, iron metabolism, or neuroinflammatory pathways. However, these CMAP-based predictions are hypothesis-generating only. Further molecular docking, ADMET analysis (especially blood–brain barrier penetration), and rigorous *in vitro* and *in vivo* validation are required before any therapeutic conclusion can be drawn. Collectively, these findings offer candidate opportunities for further exploration of pharmacological targeting of ferroptosis in AD.

Despite these insights, several limitations should be acknowledged. First, the discovery dataset was derived from brain tissue, whereas some validation datasets originated from peripheral blood, introducing heterogeneity across platforms, sample sizes, and disease stages. This likely contributed to the observed variations in effect sizes across cohorts. Additionally, in these external datasets, the model was evaluated by cohort-specific refitting rather than by applying a locked diagnostic formula, underscoring the need for prospective validation in an independent cohort. Second, as a bioinformatics-driven study, we only performed qPCR validation and did not conduct ferroptosis-specific functional assays, which are planned for subsequent research. Third, other limitations include sensitivity to WGCNA parameter choices, lack of ADMET analysis for CMAP-predicted compounds, and the modest sample size, all of which may affect generalizability.

## 5 Conclusion

This study identified three core ferroptosis-related genes, *NFKBIA*, *ATP6V1E1*, and *SUB1*, that are closely associated with Alzheimer’s disease. Their expression patterns were consistent across multiple datasets, and a predictive model based on these genes showed stable performance, supporting their potential as molecular biomarkers in AD. Mechanistic analyses suggest that these genes collectively influence neuronal vulnerability through pathways involving inflammation, oxidative stress, and iron metabolism. Pharmacogenomic prediction further highlighted compounds capable of modulating ferroptosis-related pathways, offering promising avenues for targeted intervention. Overall, this work provides novel insights into the molecular mechanisms of Alzheimer’s disease and prioritizes these candidate genes for further investigation, though prospective validation in independent cohorts is required to confirm their clinical utility.

## Data Availability

The original contributions presented in the study are included in the article/Supplementary material, further inquiries can be directed to the corresponding author/s.
